# A novel telomere-related genes model for predicting prognosis and treatment responsiveness in diffuse large B-cell lymphoma

**DOI:** 10.18632/aging.205211

**Published:** 2023-11-15

**Authors:** Zhijia Zhao, Xiaochen Shen, Siqi Zhao, Jinhua Wang, Yuqin Tian, Xiaobo Wang, Bo Tang

**Affiliations:** 1 Department of Hematology, The Second Affiliated Hospital of Dalian Medical University, Dalian 116023, People’s Republic of China; 2 Department of Pathology, The Second Affiliated Hospital of Dalian Medical University, Dalian 116023, People’s Republic of China

**Keywords:** diffuse large B-cell lymphoma, telomere-related genes, prognostic model, tumor immune environment, immune checkpoints, drug sensitivity

## Abstract

Diffuse large B cell lymphoma (DLBCL) is a highly heterogeneous disease with diverse clinical and molecular features. Telomere maintenance is widely present in tumors, but there is a lack of relevant reports on the role of telomere-related genes (TRGs) in DLBCL. In this study, we used consensus clustering based on TRGs expression to identify two molecular clusters with distinct prognoses and immune cell infiltration. We developed a TRGs scoring model using univariate Cox regression and LASSO regression in the GSE10846 training cohort. DLBCL patients in the high-risk group had a worse prognosis than those in the low-risk group, as revealed by Kaplan-Meier curves. The scoring model was validated in the GSE10846 testing cohort and GSE87371 cohort, respectively. The high-risk group was characterized by elevated infiltration of activated DCs, CD56 dim natural killer cells, myeloid-derived suppressor cells, monocytes, and plasmacytoid DCs, along with reduced infiltration of activated CD4 T cells, Type 2 T helper cells, γδ T cells, NK cells, and neutrophils. Overexpression of immune checkpoints, such as PDCD1, CD274, and LAG3, was observed in the high-risk group. Furthermore, high-risk DLBCL patients exhibited increased sensitivity to bortezomib, rapamycin, AZD6244, and BMS.536924, while low-risk DLBCL patients showed sensitivity to cisplatin and ABT.263. Using RT-qPCR, we found that three protective model genes, namely TCEAL7, EPHA4, and ELOVL4, were down-regulated in DLBCL tissues compared with control tissues. In conclusion, our novel TRGs-based model has great predictive value for the prognosis of DLBCL patients and provides a promising direction for treatment optimization.

## INTRODUCTION

Diffuse large B-cell lymphoma (DLBCL), which is the most common subtype of non-Hodgkin’s lymphoma, is a highly heterogeneous disease with diverse clinical and molecular features. Based on the cell of origin (COO) of lymphoma cells, DLBCL can be classified into germinal center B-cell (GCB) and activated B-cell (ABC) subgroups [1]. ABC DLBCL patients generally have worse prognosis. It is worth noting that DLBCL patients with similar clinicopathological characteristics have different prognoses [2]. The standard first-line therapy for DLBCL is rituximab plus cyclophosphamide, doxorubicin, vincristine, and prednisone (R-CHOP). Although R-CHOP can achieve long-term remissions in most DLBCL patients, 30–40% of them experience relapse with poor prognoses [3]. Various strategies have been explored to improve the efficacy of the standard regimen, including dose intensification, novel therapeutic agents, and next-generation anti-CD20 antibodies [4–6]. However, these approaches have not yielded significant clinical benefits [7]. Therefore, early prognosis evaluation and optimization of therapeutic options are crucial for improving the survival outcomes of DLBCL patients. The international prognostic index (IPI) is currently a widely used clinical tool for predicting the prognosis of DLBCL patients [8]. However, it has a weakness in effectively identifying prognosis of DLBCL patients with very poor survival. As the IPI score only provides prognostic information based on clinical variables, a gene expression-based signature may be a valuable supplement for further assessing the prognosis of DLBCL.

In the process of genome replication in most organisms, the main mechanism of telomere length maintenance is the completion of DNA telomere repeats by telomerase [9]. Telomerase is a ribonucleoprotein complex composed of ribonucleic acid (RNA) and protein. Its core dimer consists of telomerase reverse transcriptase (TERT) and telomerase RNA component (TERC), which is used as a template for RNA-dependent DNA synthesis [10]. Telomere maintenance is widely present in tumors and plays an important role in extending telomere length, among which telomerase activity and alternative length of telomeres (ALT) pathway are the main pathways for telomere maintenance. Among these two pathways, telomerase activation is more common and has been reported in almost all types of tumors [11, 12]. In lymphoma, Lima et al. reported that Hodgkin lymphoma (HL) cells are most likely to have telomerase activation pathways that extend telomeres, followed by the ALT pathway [13]. But there are no relevant reports in DLBCL. There are five main treatment methods for telomerase inhibition currently, including anti-hTR of oligonucleotides, nucleoside analogue, human telomerase (hTERT) small molecule inhibitor, immunotherapy for hTERT and G4 stable ligand. GRN163L (Imetelstat), one of anti-hTR of oligonucleotides, has been proven to have clinical efficacy in treating myelofibrosis and low-risk myelodysplastic syndromes [14]. Because telomeres have length abnormalities in most tumor types including lymphoma, and telomerase inhibition is currently a clear treatment method. Therefore, telomere-related genes (TRGs) should also have potential functions, prognostic judgments, therapeutic targets, and other values in DLBCL.

In this study, we comprehensively analyze the prognostic values of TRGs. Our analysis identified two distinct molecular subgroups based on expression patterns of TRGs. Notably, we developed and validated the first risk model based on TRGs in DLBCL, which demonstrated excellent predictive ability for the prognosis of DLBCL patients. By RT-qPCR, we further validated expression of candidate genes of the TRGs model, and found three of them, TCEAL7, EPHA4 and ELOVL4, were significantly lower in the DLBCL lymph node tissues and cell lines compared with normal lymph node tissues. Furthermore, we investigated the relationship between the immune infiltration, immune checkpoints and TRGs risk score, and further predicted several chemotherapy drugs that may be effective in high or low-risk DLBCL patients.

## METHODS

### Data acquisition

In this study, we obtained the mRNA expression profile and corresponding clinical information from the Gene Expression Omnibus (GEO) database. To minimize survival bias, we selected samples with a survival time greater than 0 and complete survival status information. Using this criterion, we extracted 414 samples from the GSE10846 dataset and 221 samples from the GSE87371 dataset. Additionally, we randomly divided the 414 samples in the GSE10846 dataset into a training cohort and a testing cohort at a 7:3 ratio. We used the testing cohort in the GSE10846 dataset as an internal validation cohort, and the GSE87371 dataset as an external validation cohort to verify the predictive ability of the TRG risk model. The TRGs were obtained from Telnet (http://www.cancertelsys.org/telnet/) [15], which maintains a list of genes that have been reported to be involved in telomere maintenance.

### Construction and validation of a TRGs scoring model

In this study, we utilized univariate Cox regression analysis via the R package “survival” to identify prognostic TRGs in the GSE10846 dataset. DLBCL patients were divided into 2 clusters based on different expression patterns of TRGs via the “ConsensusClusterPlus” R package. Thirty differentially expressed prognostic TRGs were found between clusters 1 and 2, and we then used the least absolute shrinkage and selection operator (LASSO) regression in the training cohort to develop a prognostic risk scoring model based on 7 TRGs. The risk scores were calculated using a formula, risk score = Σ(Expi × Coefi). We calculated risk scores in all DLBCL patients. Based on the median risk score, we divided the training cohort into low-risk and high-risk groups. We performed principal component analysis (PCA) using the “stats” package in R to explore the internal relationship between the two groups. We used Kaplan-Meier (K-M) curves to analyze overall survival (OS). To evaluate the accuracy and reliability of the risk scoring signature, time-dependent receiver operating characteristic (ROC) curve analysis was generated using the “TimeROC” package in R.

### Sample information and DLBCL cell lines

The collection of tissue samples for this study was approved by the Human Ethics Committee of the Second Hospital of Dalian Medical University, and all patients provided informed consent. We obtained nine lymph node samples from different patients at the Second Affiliated Hospital of Dalian Medical University, including two DLBCL-invaded lymph nodes from DLBCL patients and seven normal lymph nodes from abandoned tissues after surgery. Additionally, DLBCL cell lines HBL-1 and OCI-LY10 were acquired from Professor Li Li at the Second Affiliated Hospital of Dalian Medical University. HBL-1 cells were cultured in RPMI-1640 medium supplemented with 10% fetal bovine serum and 1% penicillin-streptomycin solution at 37°C with 5% CO_2_. OCI-LY10 cells were cultured in IMDM medium supplemented with 20% fetal bovine serum and 1% penicillin-streptomycin solution in a 37°C incubator with 5% CO_2_.

### Quantitative real-time PCR (RT-qPCR)

Total RNA was extracted from DLBCL cell lines or lymph node samples using Trizol reagent. Reverse transcription was performed using the PrimeScriptTMRT reagent Kit with gDNA Eraser (Takara, Japan) and RT-qPCR was performed using TB Green Premix Ex Taq™ II (Takara), according to the respective manufacturer’s instructions. The expression levels of target genes were normalized against the ACTB expression level and presented as 2^−ΔCt^. The primer sequences for RT-PCR were designed using Primer Premier 5 software and verified on BLAST websites. The primer sequences are provided in Supplementary Table 1.

### Clinical correlations and independent prognosis value of the TRGs score

The TRG scores in different clinical and pathological feature groups were performed via Wilcoxon signed-rank test and Chi-square test in the GSE10846 training dataset. Univariate and multivariate Cox regression via R package “survival” was used to evaluate the independent prognostic value of the risk score and other clinicopathological features.

### Nomogram construction

SPSS Statistics 17.0 was applied for the evaluation of collinearity and the correlation analysis of variables. Variables with strong correlations (r > |0.7|) were excluded from the subsequent analysis [16, 17]. The package “rms” of R language was applied to generate a nomogram. Area under the curve (AUC) of ROC curves was calculated using the package “survivalROC” of R language. Then, difference analysis between the AUCs of the nomogram and IPI score was conducted using Delong test via MedCalc software 20.027 for the evaluation of the nomogram’s prognostic ability and reliability [18]. In addition, decision curve analysis (DCA) was conducted, and the nomogram’s clinical utility was evaluated according to net benefits under the condition of different risk thresholds.

### Immune analyses

The single-sample Gene Set Enrichment Analysis (ssGSEA) was used to assess the enrichment of 28 immune cells and 38 immune checkpoints in the DLBCL samples, no matter between the two clusters based on differential expression patterns of TRGs, or between the high- and low-risk groups in the GSE10846 training cohort.

### Prediction of chemosensitivity

We predicted the IC50 of chemotherapeutic sensitivity for the high-risk group and low-risk group via the R package “pRRophetic”.

### Data availability statement

The datasets generated and/or analyzed during the current study are available in the GEO dataset (https://www.ncbi.nlm.nih.gov/geo/).

## RESULTS

### Molecular clustering based on the TRGs in DLBCL

**Figure 1:**
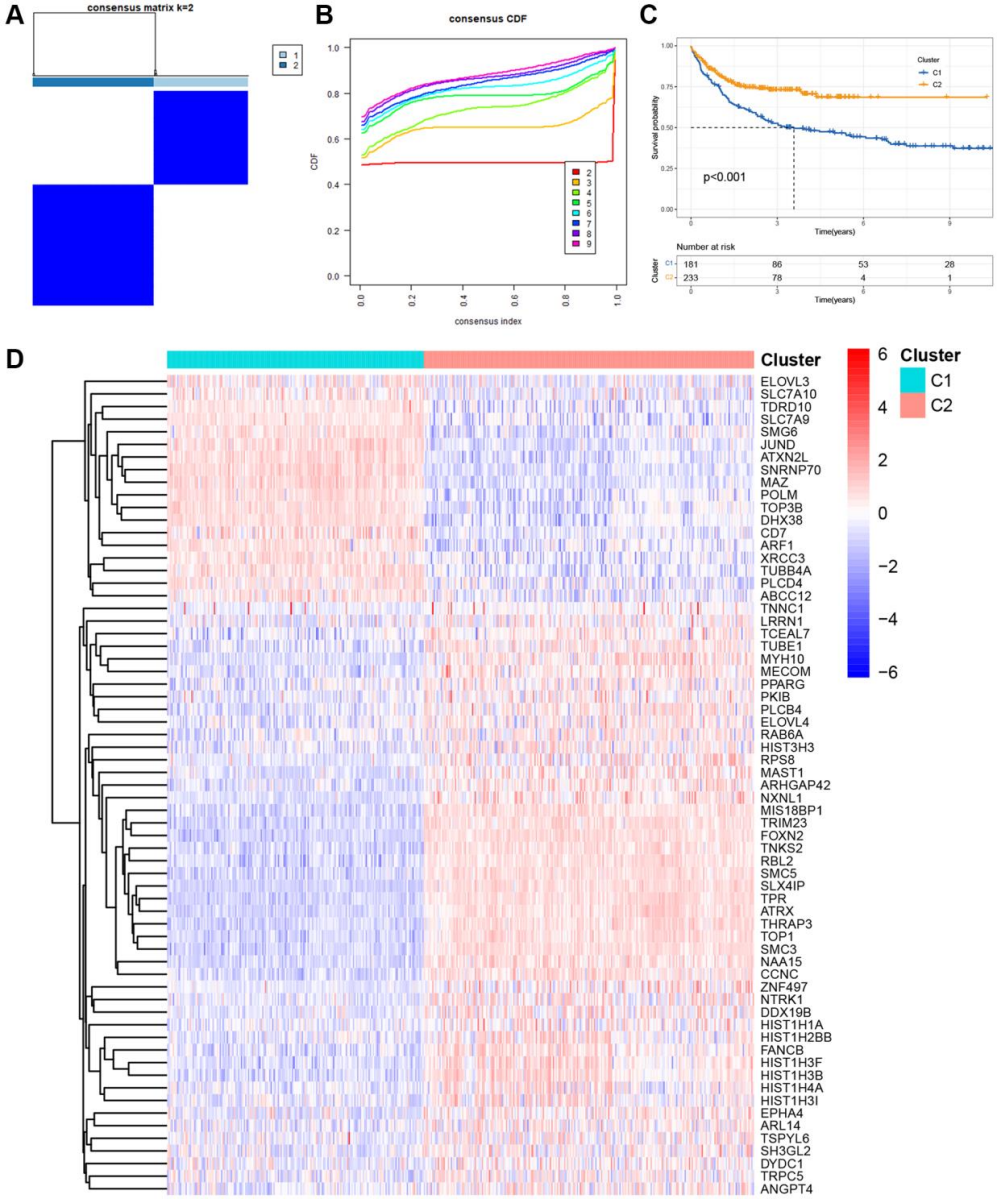
**Molecular clustering based on the TRGs in DLBCL.** (**A**) The consensus matrix by cluster analysis based on TRGs. Two clusters (k = 2) would be best. (**B**) Consensus clustering CDF with *k* value 2 in GSE10846 dataset. (**C**) Kaplan-Meier curves of OS in two clusters. (**D**) Heatmap of 65 differentially expressed genes in TRGs between clusters 1 and 2.

Initially, we retrieved 2086 TRGs from TelNet. Using univariate Cox proportional hazards regression analysis, we identified 816 prognostic TRGs that were significantly associated with the OS of DLBCL in the GSE10846 dataset (Supplementary Table 2, *p* < 0.05). Based on the expression patterns of these 816 prognostic TRGs, we performed consensus clustering to classify 414 DLBCL patients in the GSE10846 dataset into different molecular subgroups. Consensus clustering was most suitable when k = 2, as we increased the number of clusters (k) from 2 to 5 (Figure 1A, 1B and Supplementary Figure 1). Subsequently, we obtained two distinct clusters, with 181 patients in cluster 1 and 233 patients in cluster 2. K-M curves revealed that DLBCL patients in cluster 1 had significantly worse OS compared to those in cluster 2 (Figure 1C, *p* < 0.001). A heatmap illustrated 65 differentially expressed genes (DEGs) in 2086 TRGs between clusters 1 and 2 (Figure 1D).

Moreover, we investigated the differences in immune cell infiltration between these two subgroups. As depicted in Supplementary Figure 2A, activated B cells, activated CD8 T cells, activated dendritic cells (DCs), CD56 dim natural killer (NK) cells, myeloid-derived suppressor cells (MDSCs), and plasmacytoid DCs in cluster 1 exhibited higher infiltration levels than those in cluster 2. Conversely, activated CD4 T cells, gamma delta T cells, macrophages, mast cells, NK cells, neutrophils, regulatory T cells, type 17 T helper cells, type 2 T helper cells, central memory CD4 T cells, central memory CD8 T cells, and effector memory CD8 T cells in cluster 1 displayed lower infiltration levels than those in cluster 2. Additionally, we explored 38 immune checkpoint molecules between these two clusters and observed that several crucial immune checkpoint molecules, such as CD274 and PDCD1, were over-expressed in cluster 1 (Supplementary Figure 2B).

### Construction of a prognosis-associated scoring model composed of 7 TRGs

**Figure 2:**
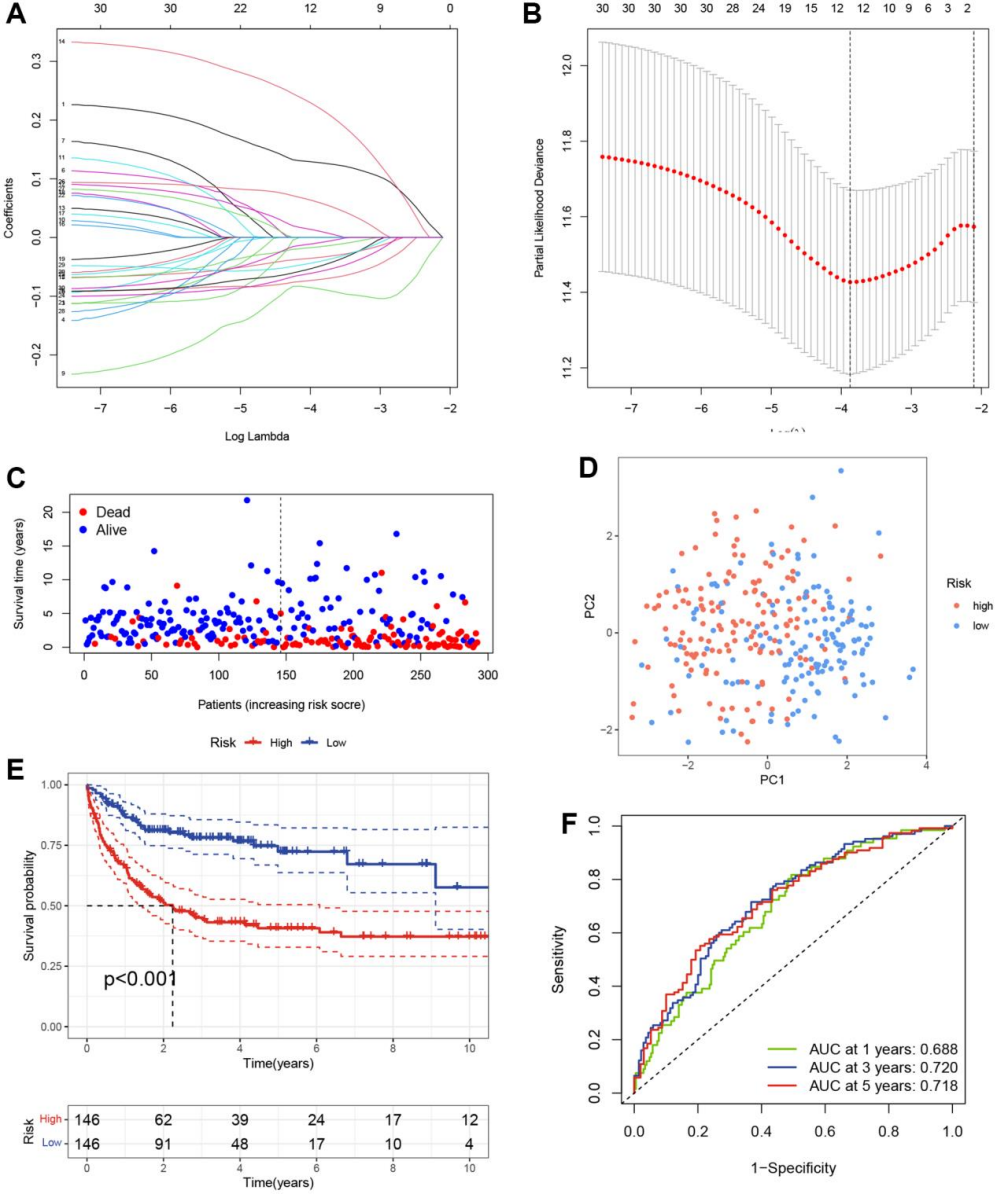
**Construction of the TRGs-based scoring model.** (**A**) LASSO coefficient profiles of TRGs in GSE10846 training cohort. (**B**) Selection of the optimal parameter (λ) in the LASSO model. (**C**) Survival status of DLBCL patients in GSE10846 training cohort. (**D**) PCA analysis of the DLBCL patients based on the TRGs score in GSE10846 training cohort. (**E**) Kaplan-Meier curves of OS based on TRGs score in GSE10846 training cohort. (**F**) Time-dependent ROC analysis of the TRGs score in GSE10846 training cohort.

Given the prognostic value of TRG expression patterns in DLBCL, we developed a TRG-based scoring model using differentially expressed TRGs between two distinct clusters. We randomly divided 414 DLBCL patients from GSE10846 into a training cohort (*n* = 292) and a testing cohort (*n* = 122) in a 7:3 ratio. The above 65 differentially expressed TRGs between clusters 1 and 2 were used to perform univariable Cox regression analysis and identify 30 TRGs associated with OS in the GSE10846 training cohort (*p* < 0.05). To avoid overfitting, we conducted LASSO regression analysis on the 30 TRGs and selected seven genes for the scoring model based on the optimal value of λ (Figure 2A, 2B). Among these genes, TUBB4A, PPARG, and ELOVL3 were identified as risk genes with HR > 1, while TCEAL7, EPHA4, ELOVL4, and ARL14 were identified as protective genes with HR < 1 (Table 1). The risk scores were calculated using the formula: risk score = (0.1873 × Exp TUBB4A) + (−0.1179 × Exp TCEAL7) + (0.2908 × Exp PPARG) + (−0.1075 × Exp EPHA4) + (−0.1001 × Exp ELOVL4) + (0.1023 × Exp ELOVL3) + (−0.0735 × Exp ARL14). Using the median risk score, we categorized DLBCL patients in the GSE10846 training cohort into high- and low-risk groups. The scatter plot revealed a worse survival outcome in the high-risk group compared to the low-risk group (Figure 2C). PCA showed a significant distribution of DLBCL patients in the high-risk and low-risk groups between two trends (Figure 2D). Furthermore, K-M curves demonstrated that DLBCL patients in the high-risk group had significantly worse OS, as illustrated in Figure 2E. We then evaluated the TRGs scoring model’s predictive efficiency using time-dependent ROC analysis, and the AUC reached 0.688 at 1-year, 0.720 at 3-year, and 0.718 at 5-year (Figure 2F).

**Table 1:** LASSO coefficient, *p*-values and hazard ratios of the seven TRGs.

**Genes**	**Coef**	**HR**	**HR.95L**	**HR.95H**	***P*-value**
TUBB4A	0.187368	1.206071	1.029736	1.412602	0.020162
TCEAL7	−0.11791	0.888775	0.791086	0.998527	0.04717
PPARG	0.290805	1.337503	1.179622	1.516516	5.69E-06
EPHA4	−0.10755	0.898033	0.781636	1.031763	0.128894
ELOVL4	−0.1001	0.904749	0.806728	1.01468	0.087103
ELOVL3	0.10239	1.107815	0.970687	1.264315	0.128841
ARL14	−0.07354	0.929102	0.843152	1.023814	0.137605

### Validation of the TRGs-based scoring model

**Figure 3:**
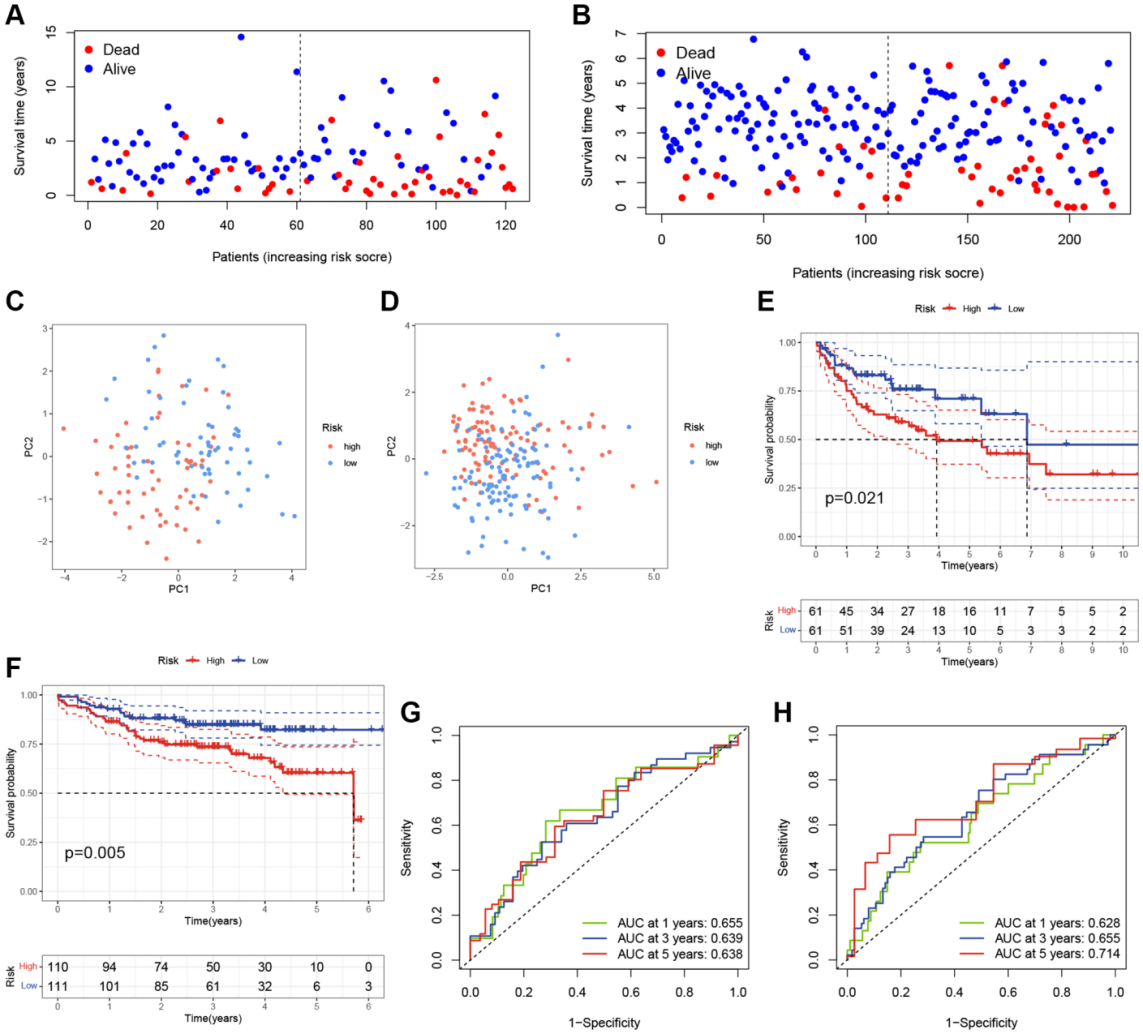
**Validation of the TRGs-based scoring model.** (**A**, **B**) The survival statuses of DLBCL patients in (**A**) GSE10846 testing cohort and (**B**) GSE87371 cohort. (**C**, **D**) The PCA of DLBCL patients in (**C**) GSE10846 testing cohort and (**D**) GSE87371 cohort. (**E**, **F**) Kaplan-Meier curves of OS based on TRGs score in (**E**) GSE10846 testing cohort and (**F**) GSE87371 cohort. (**G**, **H**) Time-dependent ROC analyses of the ARG score in (**G**) GSE10846 testing cohort and (**H**) GSE87371 cohort.

To evaluate the prediction performance and robustness of the risk model, we used the GSE10846 testing cohort as an internal validation cohort and the GSE87371 dataset as an external validation cohort. Using the same formula, we divided the patients into low- and high-risk groups based on their median risk scores in these validation cohorts. The scatter plots further revealed that patients in the high-risk group had a higher possibility of early death than patients in the low-score group in the GSE10846 testing cohort and GSE87371 cohort, respectively (Figure 3A, 3B). PCA showed that DLBCL patients in high- and low-risk groups were well-separated in validation cohorts (Figure 3C, 3D). K-M curves revealed that DLBCL patients in the low-risk group had a better prognosis than those in the high-risk group, respectively (Figure 3E, 3F). The AUC at 1-, 3-, and 5-year in the GSE10846 testing cohort were 0.655, 0.639, and 0.638 respectively (Figure 3G). In the GSE87371 cohort, the AUC reached 0.628 at 1-year, 0.655 at 3-year, and 0.714 at 5-year (Figure 3H). Overall, our prognostic TRGs-based scoring model demonstrated effective predictive efficiency in the prognosis of DLBCL patients.

### Expression evaluation of candidate TRGs in the model

**Figure 4:**
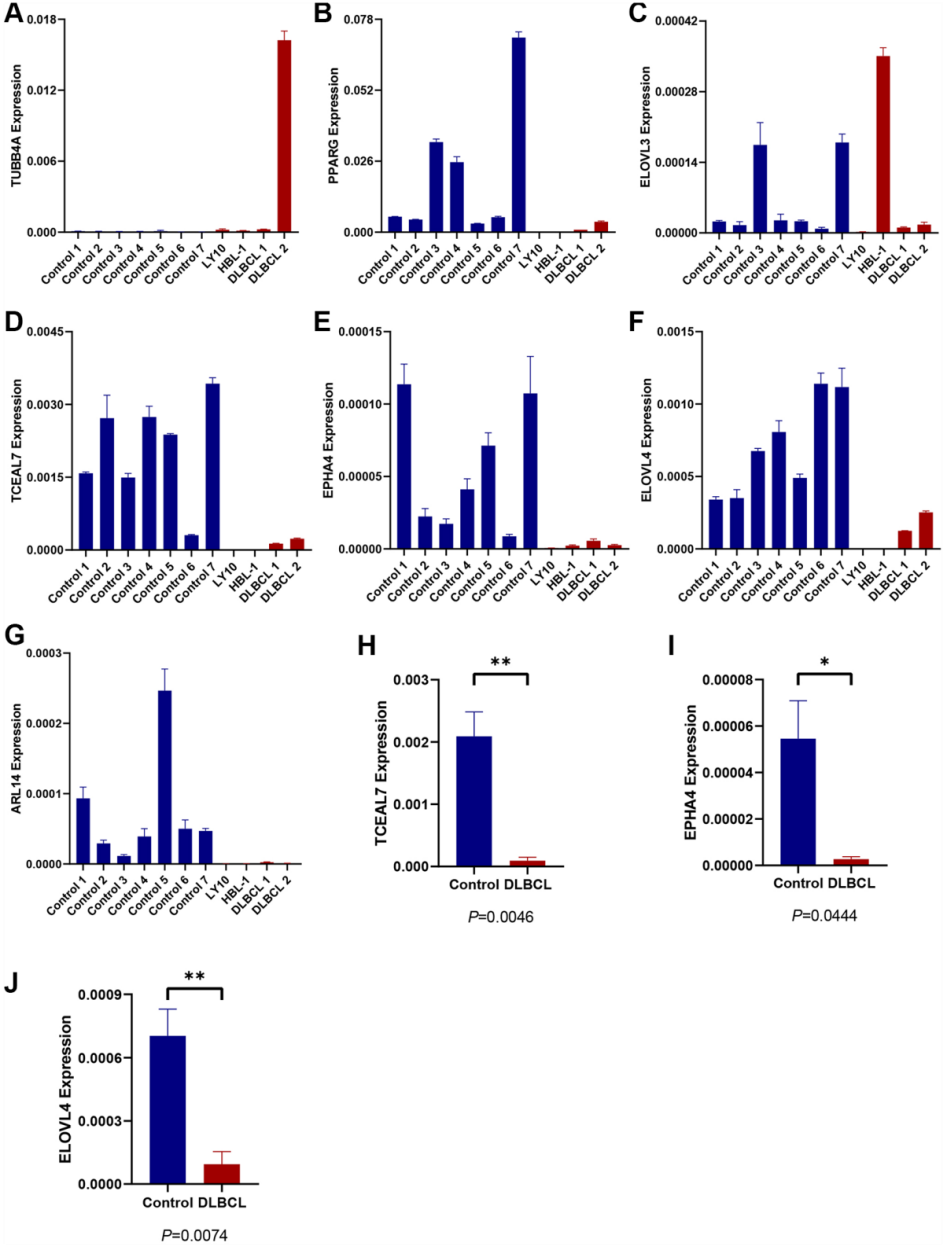
**Expression evaluation of candidate TRGs in the model.** (**A**–**G**) The expression level of 7 TRGs in two DLBCL cell lines (HBL-1 and OCI-LY10), two DLBCL-invaded lymph node samples (DLBCL 1 and DLBCL 2) and seven normal lymph node samples (control 1–7), respectively. (**H**, **I**) The expression of (**H**) TCEAL7, (**I**) EPHA4, and (**J**) ELOVL4 in DLBCL group and control group.

To validate the expression of 7 candidate model genes in DLBCL patients, we collected samples from two DLBCL cell lines (HBL-1 and OCI-LY10), two DLBCL-invaded lymph nodes, and seven normal lymph nodes. RT-qPCR analysis revealed that the expression levels of several protective genes, TCEAL7, EPHA4, and ELOVL4, were significantly lower in the DLBCL group than in the control group (Figure 4A–4J). There was no statistically significant difference in the expression of the other four candidate genes between the DLBCL and control groups based on the current sample sizes (Supplementary Figure 3).

### Clinical correlations and independent prognosis value of TRGs risk model

**Figure 5:**
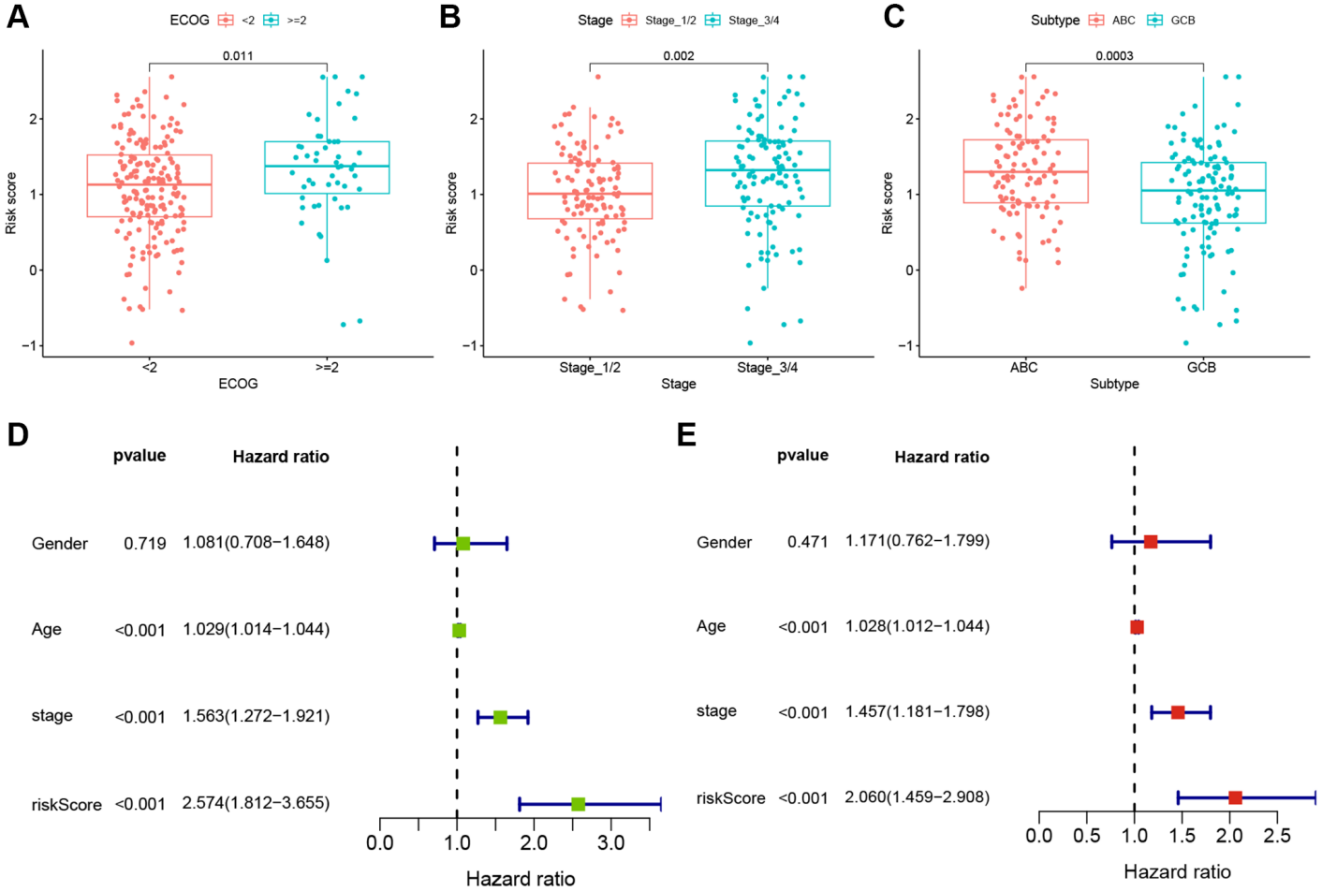
**Clinical correlations and independent prognosis value of TRGs risk model.** (**A**–**C**) TRGs risk scores in different DLBCL subgroups of (**A**) EOCG, (**B**) stage and (**C**) subtype. (**D**) Univariate Cox regression analysis of TRGs score and clinical features in GSE10846 training cohort. (**E**) Multivariate Cox regression analysis of TRGs score and clinical features in GSE10846 training cohort.

To evaluate the clinical significance of the TRGs risk score, we compared the risk scores between different subgroups based on clinical features in the GSE10846 training cohort. As shown in Figure 5A–5C, the risk scores were significantly higher in DLBCL patients with ECOG ≥ 2, stage 3–4, or ABC subtype.

To explore whether TRG risk score could be an independent prognostic factor, we first used univariate Cox regression analysis to evaluate it and other clinical characteristics (age, gender and stage) in these cohorts. Age, stage and TRGs risk score were related to the OS of DLBCL patients in the GSE10846 training cohort (all *p*-values < 0.001, Figure 5D). In the GSE10846 testing cohort, age and TRGs risk score were highly associated with the prognosis of DLBCL patients (all *p*-values < 0.05, Supplementary Figure 4A). In the GSE87371 cohort, age, stage and TRGs risk score had close relationships with OS (all *p*-values < 0.001, Supplementary Figure 4B). Furthermore, we conducted multivariate Cox regression analysis to adjust for confounding factors. TRG risk score was found to be an independent predictor of OS in all of the 3 cohorts (Figure 5E and Supplementary Figure 5A, 5B; GSE10846 training cohort: HR = 2.060, *p* < 0.001; GSE10846 testing cohort: HR = 1.740, *p* = 0.036; GSE87371 cohort: HR = 4.359, *p* = 0.014).

### Nomogram construction

**Figure 6:**
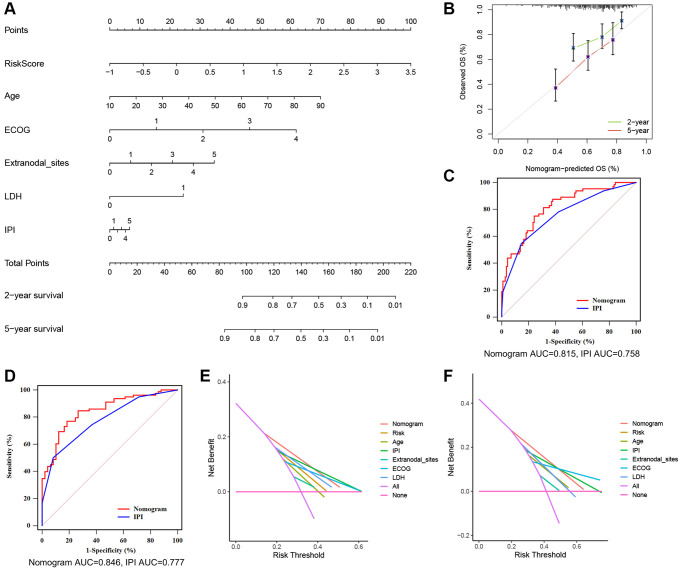
**A nomogram based on the TRGs risk score, IPI score and clinical features.** (**A**) A nomogram for the prediction of DLBCL patients’ 2- and 5-year survival probability according to the TRGs score, IPI score and clinical factors. (**B**) Nomogram-predicted percentages and the observed probabilities of 2- and 5-year survival. (**C**, **D**) Time-dependent ROC analysis of the nomogram and IPI score at 2- (**C**) and 5-year (**D**) survival. (**E**, **F**) DCA of the constructed nomogram compared with different indicators at 2- (**E**) and 5-year (**F**) survival.

Nomograms are useful tools for predicting patient prognosis and can assist with clinical decision-making. After the evaluation of collinearity and the correlation analysis of variables (Supplementary Tables 3 and 4), we constructed a prognostic nomogram based on the TRGs risk score and clinical features, including age, ECOG, LDH level, number of extranodal sites and IPI score (Figure 6A). The nomogram showed excellent consistency between predicted and observed OS at 1-year, 3-year, and 5-year, as demonstrated by the favorable match of the calibration curves (Figure 6B). We further compared the ROC curves of the nomogram with the IPI score and found that the AUC of the nomogram was higher than that of the IPI score at 2- and 5-year survival outcome (2-year 0.815 vs. 0.758 and 5-year 0.846 vs. 0.777, Figure 6C, 6D). Subsequently, difference analysis (Table 2) presented statistical differences between the AUCs of nomogram and IPI score at 2- and 5-year survival. Meanwhile, DCA curves were made for the comparations of predicted net benefit between the nomogram and IPI score, and the findings reflected the certain feasibility of this constructed for making valuable prognostic judgments and therapeutic guidance. As shown in Figure 6E, 6F, when the risk threshold of patients was approximately 15–55% at 2-year survival or 20–65% at 5-year survival predicted by the nomogram, the nomogram application for therapeutic guidance would provide more benefit than either treating all patients or employing no treatment. Additionally, DCA curves showed that the nomogram presented superior benefits compared with IPI score in the condition of 15–35% threshold probability at 2-year survival or 20–45% threshold probability at 5-year survival. These results demonstrate that the nomogram has better ability to forecast the OS of DLBCL than the IPI score.

**Table 2:** Difference analysis of AUCs between the nomogram and IPI.

	**2-year survival**	**5-year survival**
Difference between AUCs	0.057	0.0691
z statistic	2.211	2.444
*p*-value	0.0270	0.0145

### Correlation between risk score and immune microenvironment

**Figure 7:**
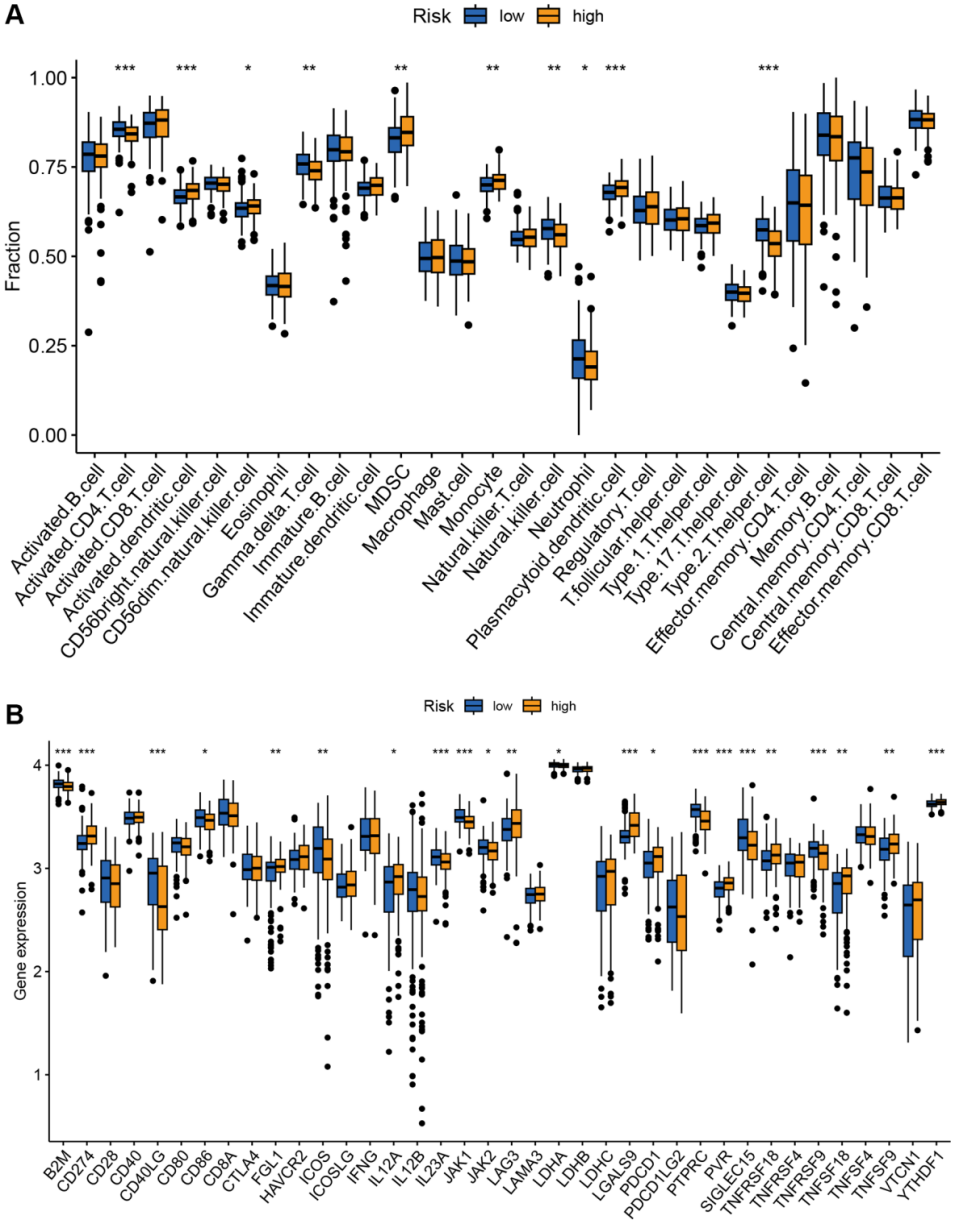
**Correlation analysis of TRGs risk score with immune landscape.** (**A**) The proportion of 28 immune cells between low-risk and high-risk groups (^*^p < 0.05; ^**^p < 0.01; and ^***^p < 0.001). (**B**) The distribution of 38 immune checkpoints between the high-risk and low-risk groups (^*^p < 0.05; ^**^p < 0.01; and ^***^p < 0.001).

The tumor immune microenvironment plays a crucial role in the therapeutic response and prognosis of tumors. Therefore, we analyzed the immune infiltration of high- and low-risk groups in the GSE10846 training cohort. The ssGSEA revealed higher levels of infiltrating activated DCs, CD56 dim NK cells, MDSC, monocytes, and plasmacytoid DCs in the high-risk group. Additionally, lower levels of infiltrating activated CD4 T cells, Type 2 T helper cells, γδ T cells, NK cells, and neutrophils were observed in the high-risk group (Figure 7A). Furthermore, expression differences in most of the immune checkpoints were found between the high-risk and low-risk groups. Higher expression levels of PDCD1, CD274, LAG3, FGL1, LGALS9, PVR, TNFRSF18, TNFSF18, YTHDF1, IL12A, and TNFSF9 were observed in the high-risk compared to the low-risk group. Conversely, expression of B2M, CD40LG, CD86, ICOS, IL23A, JAK1, JAK2, LDHA, PTPRC, SIGLEC15, and TNFRSF9 were downregulated in the high-risk group (Figure 7B).

### The risk signature predicted the sensitivity of novel chemotherapy

**Figure 8:**
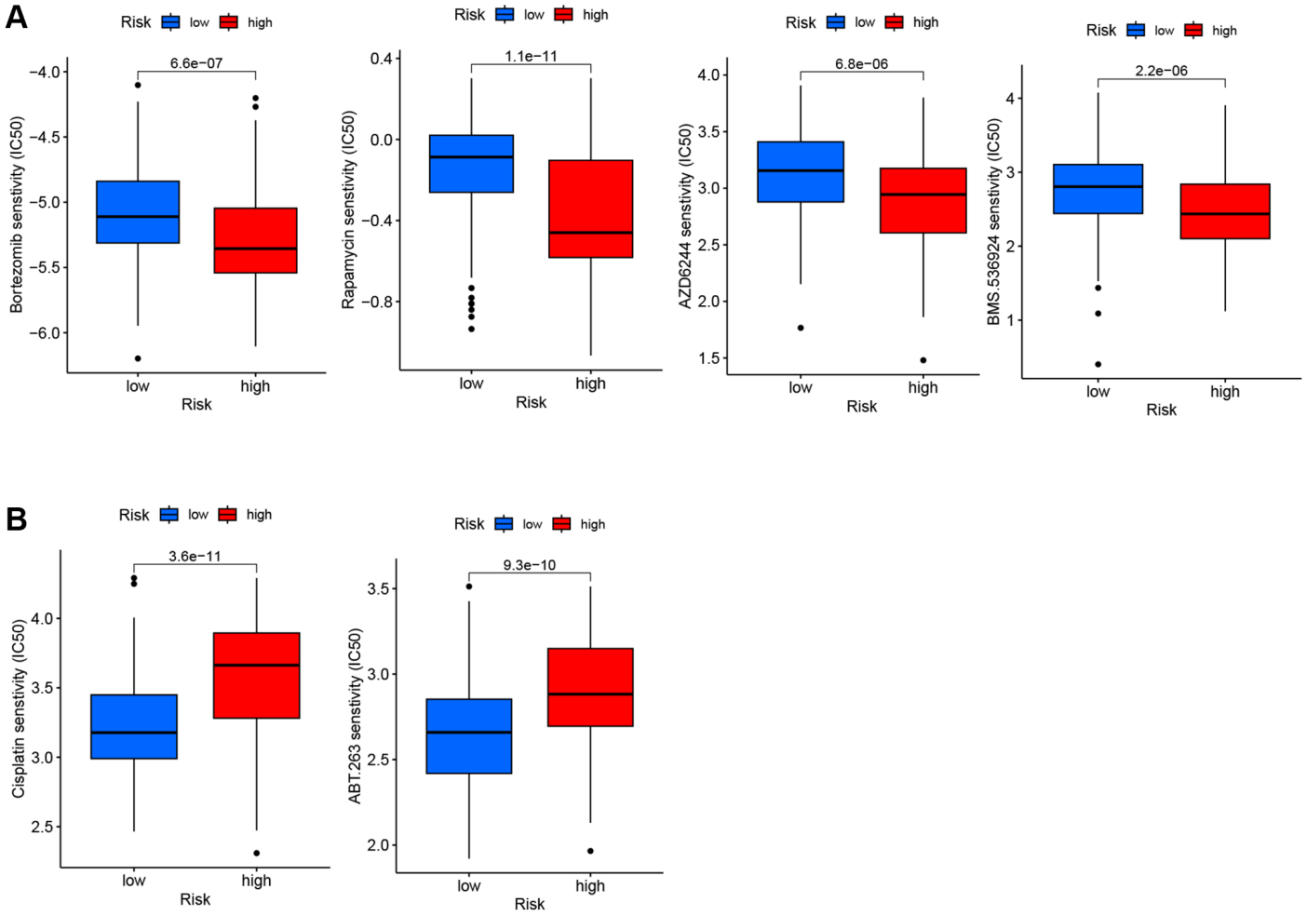
**IC50 of six drugs between low-risk and high-risk groups.** (**A**) The high-risk group exhibited more sensitivity to the four drugs compared to the low-risk group. (**B**) The high-risk group exhibited less sensitivity to the two drugs compared to the low-risk group.

We estimated the IC50 values of different drugs in the GSE10846 dataset to explore whether the TRG score presented a potential association with drug sensitivity. Compared to the low-risk group, the high-risk group exhibited increased sensitivity to bortezomib, rapamycin, AZD6244 (MEK1/2 inhibitor), and BMS.536924 (IGF-1R inhibitor) (Figure 8A). DLBCL patients in the low-risk group showed sensitivity to cisplatin (a drug used in several DLBCL second-line chemotherapy regimens) and ABT.263 (Bcl-2 inhibitor, Figure 8B).

## DISCUSSION

Chemotherapy and immunotherapy have played principal roles in the treatment of DLBCL patients, but the overall survival rate of DLBCL remains disappointing due to phenotypic heterogeneity and high relapse rate. Dividing patients into high-risk or low-risk groups to evaluate prognosis and drug reaction can help doctors increase accuracy and personalize clinical treatment. Telomere maintenance is widely present in tumors and plays an important role in extending telomere length, which can lead to immortalization and uncontrolled proliferation [19]. However, the relationship between TRGs and prognosis of DLBCL is rarely reported. Furthermore, there has been no report about TRG-based scoring model in DLBCL available.

To the best of our knowledge, our study is the first to investigate the correlation between TRGs and the prognosis of DLBCL. Using consensus clustering, we identified two subtypes with significant differences in prognosis. Compared to cluster 2, the prognosis of DLBCL patients in cluster 1 was poor. Because expression patterns of TRGs can effectively distinguish the prognosis of DLBCL patients, we further construct a prognostic TRGs scoring model. Among the candidate model genes, TUBB4A, PPARG and ELOVL3 served as risk genes with HR > 1, and TCEAL7, EPHA4, ELOVL4 and ARL14 were protective genes with HR < 1.

Previous studies have expounded the specific roles of the candidate TRGs in different tumors. As one hot spot of the current research, PPARG encodes PPARγ, one of representative nuclear receptors. PPARγ is related to the pathology of many diseases, including obesity, diabetes, atherosclerosis and cancer, and notably, the effects of PPARγ in the tumor occurrence and development are not unifying [20]. PPARγ functions as a tumor-suppressive factor via PPARγ/RXRα signaling pathway in several tumor types [21]. Contrarily, it has been reported that the activation of PPARγ/RXRα induces the microenvironmental reprogramming in the bladder cancer, which is beneficial to the tumorigenesis [22]. In addition to those solid tumors, the function of PPARG has been also studied in lymphoma. As one of oxidative stress genes, PPARG increases the generation of reactive oxygen species appears to increase the risk for non-Hodgkin lymphoma, particularly DLBCL [23]. Elevated expression of PPARγ increases expression of fatty acids to inhibit NK cell response and cell metabolism in invasive B-cell lymphoma, which leads to the functional adaptation of NK cells to fatty acid rich lymphoma [24]. EPHA4 belongs to the largest RTK-Eph family. Some studies have shown that RTKs play an important role in Epstein-Barr virus (EBV)-related tumor formation, because deceased expression of EPHA4 is associated with EBV infection. EBV is an important pathogenic factor for lymphoma and is closely related to the pathogenesis of some DLBCL [25]. Virus-related lymphoid malignancies activate telomerase early via the exogenous regulator of hTERT [26]. These findings seem to indicate that reduced expression of EPHA4 may cause lymphoma through EBV infection. Corresponding to the above findings, overexpression of EPHA4 prevented proliferation of lymphoblastoid cell lines [27]. According to the scoring TRGs model we constructed, EPHA4 was identified as a protective gene and its low-expression was related to poor survival in DLBCL. In the combination of low EPHA4 expression confirmed via RT-qPCR, the tumor-preventing role and prognostic value of EPHA4 in DLBCL become more convincing. Besides, EPHA4 may be applied in the molecular diagnosis of Sézary syndrome-related cutaneous T-cell lymphomas, and the membrane-bound EPHA4 receptor can serve as a target for targeted therapeutic interventions [28]. Among other five TRGs, TUBB4A serves as a tumor-promoting factor in several tumor types including melanoma and prostate cancer, and ELOVL3 is considered as a risk gene in hepatocellular carcinoma [22, 29, 30]. However, there has been no relevant report in lymphomas. Moreover, TCEAL7 is a competitive inhibitor of c-Myc, and c-Myc plays an important role in telomere maintenance mechanisms as a hTERT transcriptional activator [31, 32]. Studies show that TCEAL7 is down-regulated in many tumors, and is considered as a putative tumor suppressor gene. Consistent with the conclusions made by others, TUBB4A and ELOVL3 presented as risk genes, while down-regulation of TCEAL7 was confirmed in DLBCL and correlated with poor prognosis in our study. As for ELOVL4, the effects in tumors remain controversial. It has been reported that ELOVL4 serves as a tumor suppressor via the NOTCH-RIPK4-IRF6-ELOVL4 axis in squamous cell carcinoma, and its overexpression is related to the good prognosis for neuroblastoma patients [33, 34]. On the contrary, ELOVL4 is identified as a risk gene in gastric cancer [35]. Moreover, ARL14 has only been reported in non small-cell lung cancer (NSCLC), and increased expression of ARL14 was significantly correlated with poor survival [36]. However, ARL14 was considered as a protective gene in our study, differing from the function observed in NSCLC. We considered that the difference may be related to the variations of tumor tissue origins and tumor microenvironment (TME). Therefore, more experimental evidence is still needed to identify the respective functions of these TRGs in DLBCL.

TRG risk model was not only validated in the training dataset, but also in the external validation set. The AUC values of the ROC curves in our training dataset for 1-, 3-, and 5-year OS were 0.688, 0.720, and 0.718, 1-, 3-, and 5-year OS were 0.655, 0.639 and 0.638 in internal validation set, and 1-, 3-, and 5-year OS were 0.628, 0.655 and 0.714 in external validation set, respectively. These results indicate that TRG risk model is a stable and reliable model for evaluating the prognosis of DLBCL. A nomogram that integrates TRG risk score, IPI score and other clinical features such as age, ECOG status, extranodal sites, and LDH level further provides the possibility of individualized utility in predicting patient prognosis.

In our study, high level of MDSC and low levels of CD4 T cells, NK cells and γδ T cells were found in the TME of high-risk patients. The abundance of NK cells predicted better outcomes in DLBCL patients, which may be attributed to the fact that high NK cell count enhances the efficacy of R-CHOP [37]. Studies have shown that MDSCs suppress the immune response to promote the occurrence and development of tumors in tumor microenvironment [38]. For example, a reduction in the number of MDSCs in patients with metastatic breast cancer treated with the drug cabozantinib was related to improved progression-free survival [39]. Recent evidence also has indicated that the presence of activated CD4 T cells in tumor tissue is associated with a better prognosis [40]. Besides, γδ T cells showed synergistic anti-tumor effects with activated αβ T cells and NK cells which improved patient outcomes [41, 42].

Tumor immune escape is considered as one of vital mechanisms in tumor occurrence and progression, and one of its main factors is the regulation of immune checkpoint expression [43]. The therapy targeting immune checkpoints has been a hot direction in the current clinical research and is viewed as one of effective antitumor therapies. In our study, several important immune checkpoints were found overexpressed in the high-risk group, such as PDCD1 and CD274. CD274, also known as programmed cell death ligand 1 (PD-L1), is a ligand for the inhibitory receptor PDCD1/PD-1, which regulates the activation threshold of T cells and limit T cell effector responses. The interaction between PD-L1 and PDCD1/PD-1 is a way to reduce anti-tumor immunity and evade immune system damage, thereby promoting tumor survival. Blocking the PD-1/PD-L1 pathway can normalize anti-tumor responses, but PD-1 inhibitors are not recommended to treat unselected DLBCL patients, due to low expression of PD-1/PD-L1 in DLBCL. Our results revealed that the high-risk group had significantly higher expression levels of CD274 and PDCD1, which contributed to identifying DLBCL patients who are susceptible to PD-1 inhibitors [44–46]. Our study also showed that expression of JAK1 and JAK2 in high-risk group was lower than that in the low-risk group. The downstream target protein STAT of the JAK family is an important cytokine activated transcription unit in the immune response. The sustained activation of STAT3 and STAT5 can increase tumor cell proliferation and disease progression, it would be therapeutic targets for enhancing anti-tumor immunity [47, 48]. Activated STAT3 can drive the dissemination of DLBCL [49]. Related drugs, such as Cerdulatinib, is a novel dual SYK/JAK kinase inhibitor and has broad anti-tumor activity in DLBCL [50]. Therefore, treatment of the JAK pathway may have better efficacy in the low-risk groups.

In addition, due to the relationship between high-risk score and poor prognosis, the correlation between chemotherapy resistance and risk score was further studied. We found that the IC50 of bortezomib, rapamycin, AZD6244 and BMS-536924 in the high-risk group were lower than those in the low-risk group, suggesting that the high-risk group was more sensitive to these drugs. Bortezomib is a proteasome inhibitor that acts on the NF-ĸB signaling pathway, which is increased in non-GCB DLBCL. Combining bortezomib with R-CHOP in the treatment of recurrent non-GCB DLBCL is better than that of GCB DLBCL, and can improve the prognosis of non-GCB DLBCL [51]. Rapamycin is an mTOR inhibitor that can enhance the cytotoxicity induced by rituximab. Combined use of rapamycin may improve the efficacy of DLBCL patients [52]. AZD6244, a MEK inhibitor, has been shown to downregulate ERK substrate related to DLBCL cells and induce cell apoptosis [53]. BMS-536924 is a dual IGF1R/IR kinase inhibitor that has been experimentally proven to inhibit acute myeloid leukemia (AML) proliferation and serve as a potential therapeutic target for AML [54]. In the low-risk group, the IC50 of ABT-263 and cisplatin were lower than that of the high-risk group, indicating that they were sensitive to it. ABT-263 is one of the inhibitors of the Bcl-2 family. Experiments have shown that ABT-263 combined with a variety of chemotherapy drugs can inhibit and delay the growth of hematologic malignancies [55]. Cisplatin is a commonly used chemotherapy drug in hematological tumors. For patients with refractory relapse-resistant DLBCL who can tolerate high-dose chemotherapy, platinum-based regimen is the most commonly used, such as DHAP (dexamethasone, cisplatin and cytarabine) [56].

In our study, this scoring model was validated in both of the training dataset and the external validation set. When the nucleotide sequences of tumor samples from biopsies are confirmed and analyzed via Next Generation Sequencing of the same platform, the score can be calculated. Combining the above results of predicted chemotherapeutic sensitivity based on the scoring model, a new therapeutic strategy for DLBCL patients with different risk was formed, that is applying bortezomib, rapamycin, AZD6244 and BMS-536924 in the high-risk group, and cisplatin-containing regimens in the second-line treatment of relapse-refractory DLBCL patients in the low-risk group. Additionally, our study considered this scoring model as an indicator integrated in the constructed nomogram, and the subsequent DCA curves showed the positive net benefits of the constructed nomogram in guiding clinical decisions.

Nevertheless, there exist some limitations. Our study applied training dataset GSE10846 and validation dataset GSE87371 for the construction and verification of the scoring model. Although the detection platform is the same across both two datasets, the laboratories involved and their respective countries differ from each other. Therefore, differences in experimental operations (conducted by different individual experimenters), handling and exposure time of tumor samples prior to RNA extraction and inconsistent reagents occurs, causing discrepancies in our analysis.

Based on the above findings, we developed a TRGs-based scoring model for DLBCL, which represents a novel approach in this field. Our model has the potential to accurately predict the prognosis of DLBCL patients and facilitate the development of personalized treatment strategies.

## Supplementary Materials

Supplementary Figures

Supplementary Tables
