# Supplementary material for: A novel telomere-related genes model for predicting prognosis and treatment responsiveness in diffuse large B-cell lymphoma
Source: Aging (Albany NY). 2023 Nov 15;15(22):12927–51. doi: 10.18632/aging.205211 (PMC10713390; doi:10.18632/aging.205211)
Supplement: Supplementary Figures [file aging-15-205211-s001.pdf]

SUPPLEMENTARY FIGURES

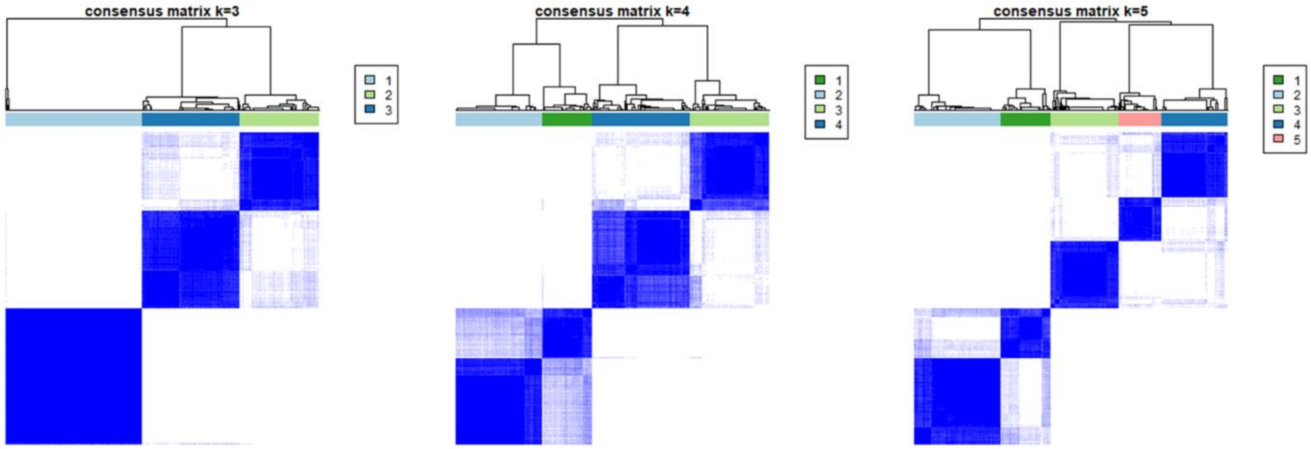

Supplementary Figure 1. The consensus matrix by cluster analysis based on TRGs. TRGs-based consensus clustering heatmap from 412 DLBCL samples of GSE10846 (k = 3−5).

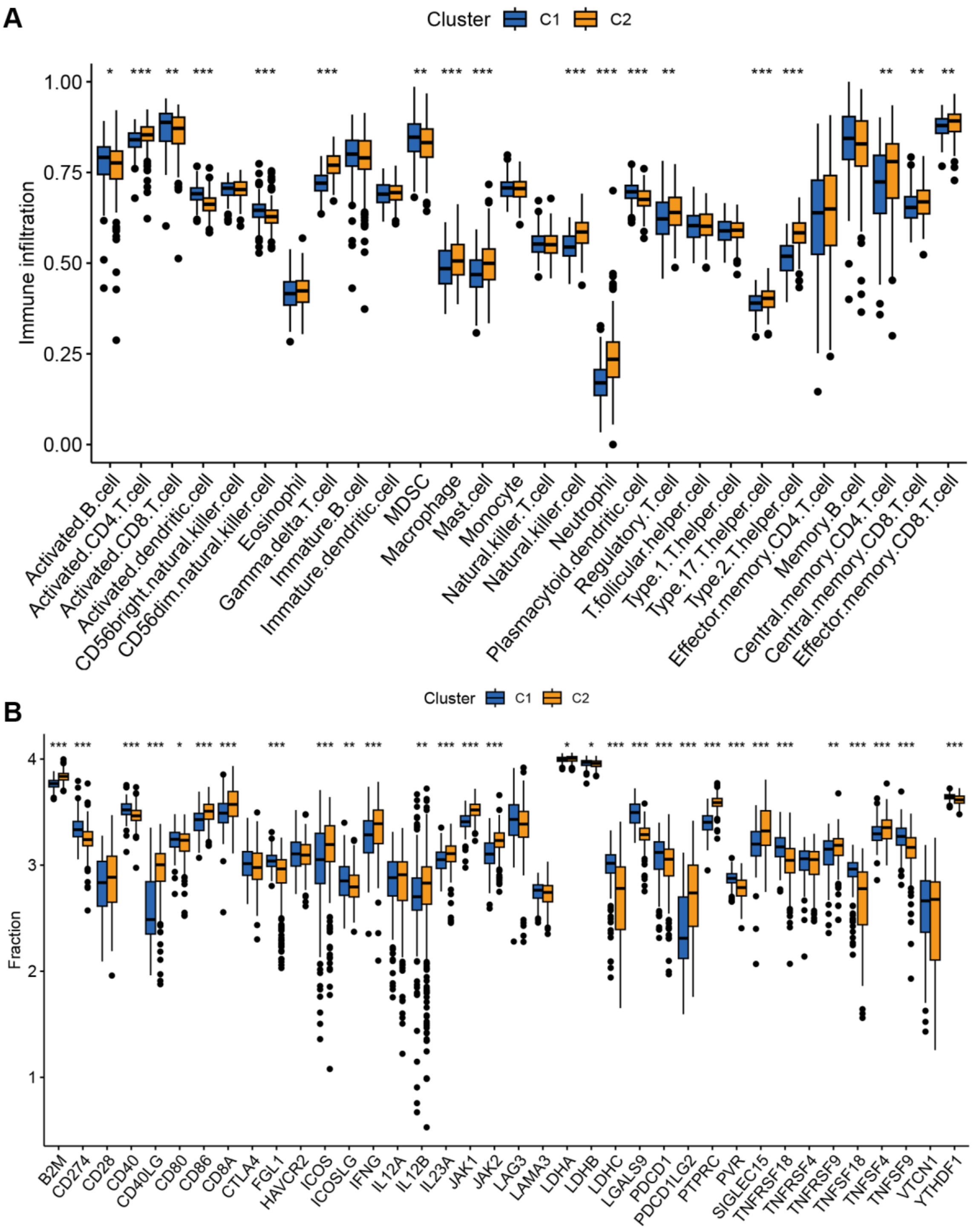

Supplementary Figure 2. Different immune profiles between two clusters. (**A**, **B**) The differences of (**A**) 28 immune cells and (**B**) 38 immune checkpoints between two clusters by ssGSEA ($^{*}p < 0.05$; $^{**}p < 0.01$; and $^{***}p < 0.001$).

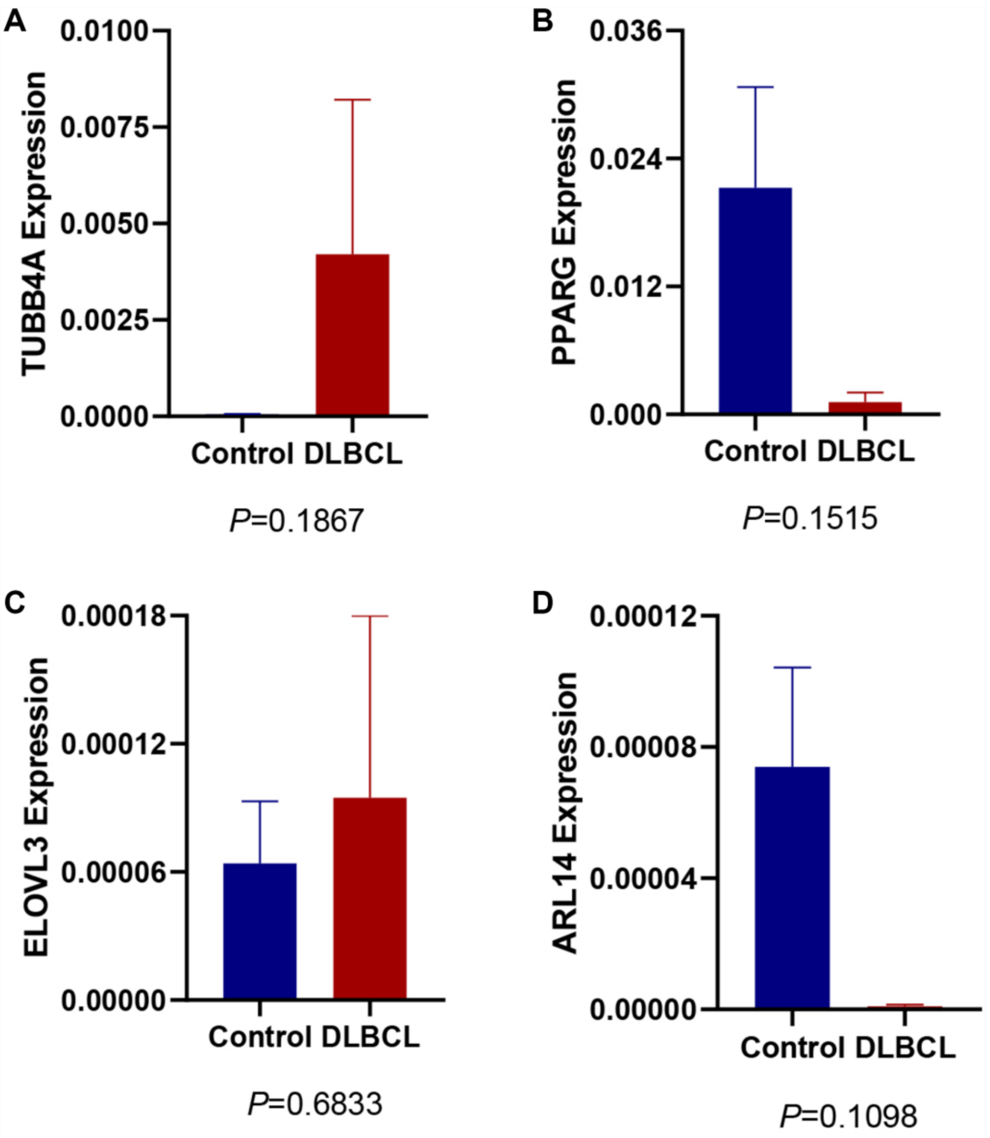

Supplementary Figure 3. Expression evaluation of other candidate TRGs in the model. (**A**–**D**) The expression of (**A**) TUBB4A (**B**) PPARG, (**C**) ELOVL3 and (**D**) ARL14 in DLBCL group and control group.

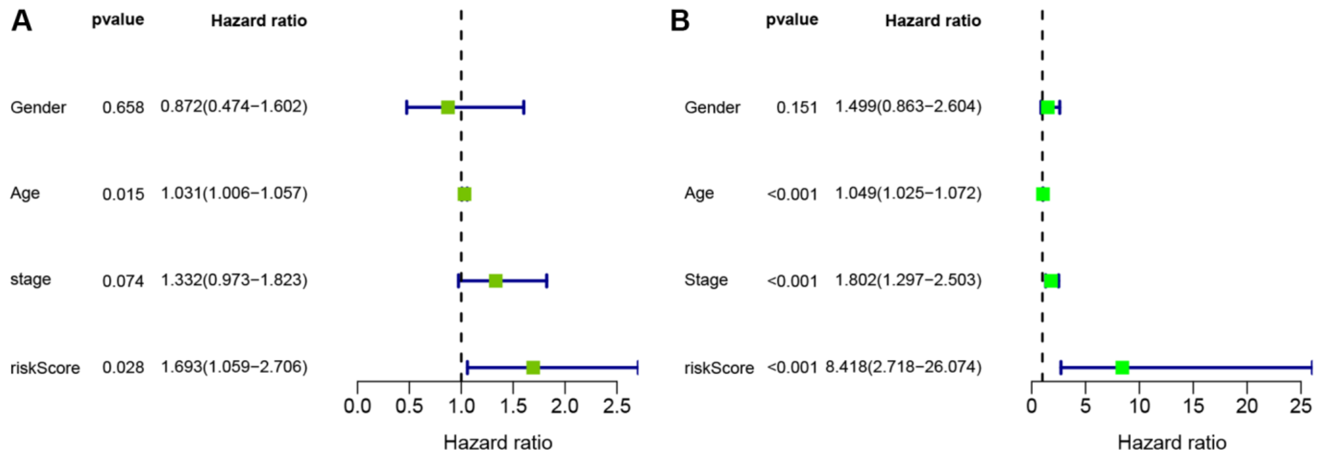

Supplementary Figure 4. Univariate Cox regression analysis of the TRGs score and clinical features. (**A**, **B**) Univariate Cox regression analysis of TRGs score and clinical features in (**A**) GSE10846 testing cohort and (**B**) GSE87371 cohort.

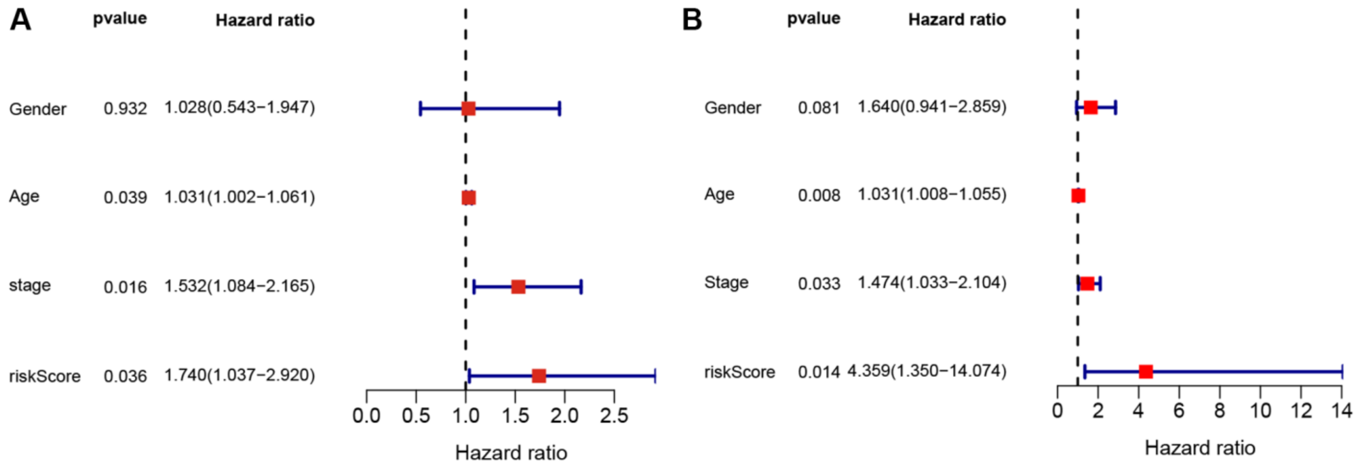

Supplementary Figure 5. Multivariate Cox regression analysis of the TRGs score and clinical features. (**A**, **B**) Multivariate Cox regression analysis of TRGs score and clinical features in (**A**) GSE10846 testing cohort and (**B**) GSE87371 cohort.
