# Supplementary material for: A novel telomere-related genes model for predicting prognosis and treatment responsiveness in diffuse large B-cell lymphoma
Source: Aging (Albany NY). 2023 Nov 15;15(22):12927–51. doi: 10.18632/aging.205211 (PMC10713390; doi:10.18632/aging.205211)
Supplement: Supplementary Tables [file aging-15-205211-s002.pdf]

SUPPLEMENTARY TABLES

Supplementary Table 1. The primer sequences of candidate genes and one reference gene (ACTB) for RT-PCR.

Primer	Sequence
TUBB4A Forward	5′–3′ CCGGACAACTTCGTGTTTGG
TUBB4A Reverse	5′–3′ TCGCGGATCTTACTGATGAGC
PPARG Forward	5′–3′ ACCAAAGTGCAATCAAAGTGGA
PPARG Reverse	5′–3′ ATGAGGGAGTTGGAAGGCTCT
ELOVL3 Forward	5′–3′ CTGTTCCAGCCCTATAACTTCG
ELOVL3 Reverse	5′–3′ GAATGAGGTTGCCCAATACTCC
TCEAL7 Forward	5′–3′ AAGGGAAGGAAGAGGTCCCAG
TCEAL7 Reverse	5′–3′ CTCTGTGCGGGGTAGTTTCC
EPHA4 Forward	5′–3′ TTCGCCCTATTTTCGTGTCTC
EPHA4 Reverse	5′–3′ TGGTAGGTTCGGATTGGTGTAT
ELOVL4 Forward	5′–3′ GAGCCGGGTAGTGTCCTAAAC
ELOVL4 Reverse	5′–3′ CACACGCTTATCTGCGATGG
ARL14 Forward	5′–3′ AAATCCGCAAACCAAACAAGC
ARL14 Reverse	5′–3′ TTCCAACTCGATCATTTCCACAT
ACTB Forward	5′–3′CCTGGCACCCAGCACAAT
ACTB Reverse	5′–3′GCCGATCCACACGGAGTA

Supplementary Table 2. Prognostic telomere-related genes.

ZSWIM7	THRAP3	RPS7	POLM	NCL	JSRP1	FANCB	CEP85	AHR
ZNHIT3	THOC6	RPS6KA5	POLE3	NCDN	JAZF1	FAM50A	CEP164	ACY1
ZNF703	THOC2	RPS4X	POLE2	NCBP2	JAK2	FAM175A	CENPO	ACTR5
ZNF414	THOC1	RPS28	POLDIP3	NCAPD3	IWS1	EZH1	CEBPA	ACTR3
ZNF281	THEMIS2	RPS17	POLD4	NBN	IVD	EXOSC6	CDKN2B	ACTR2
ZNF148	TFPT	RPS16	POLD2	NAT16	ITGAX	EXO1	CDK9	ACTN1
ZNF140	TFEC	RPS15A	POLD1	NAT10	ISCA2	ETAA1	CDK19	ACLY
ZMYM2	TERT	RPS14	POC1A	NASP	IRS4	ESRRA	CDK18	ABCF1
ZMIZ2	TERF1	RPN1	PNPO	NAGS	IRAK1	ESR2	CDK16	ABCC5
ZGPAT	TENM3	RPLP2	PNKP	NADK	IPO13	ERMP1	CDK13	ABCC4
ZCCHC7	TELO2	RPLP1	PNKD	NACA	IP6K3	ERH	CDK12	ABCC12
ZC3H18	TEAD1	RPL31	PMS1	MZF1	INO80E	ERCC5	CDK10	ABCC10
ZBTB9	TDRD7	RPL26L1	PLOD2	MYOM2	INO80C	ERCC4	CDCA8	ABCC1
ZBTB48	TCOF1	RPL19	PLK1	MYO10	INO80	ERCC3	CDC73	ABCB7
ZBTB44	TCF7L2	RPAP3	PLCL1	MYH9	ILF3	ERCC2	CDC45	AARS2
YY1	TCF7	RPAP1	PLCD1	MYH10	ILF2	EPS8L1	CDC37	AARS
YWHAQ	TCEAL7	RPAIN	PLCB4	MYC	IFRD2	EPHX2	CDC27	
YWHAG	TBCA	RPA3	PLCB3	MVK	IDH3A	EPHA4	CDC25B	
YWHAE	TBC1D15	RPA2	PLCB2	MUTYH	HYOU1	ENAH	CDC16	
YWHAB	TARDBP	RNF40	PLAT	MTF2	HUS1	EME1	CCT4	
YTHDF3	TALDO1	RINT1	PKMYT1	MTA1	HSPA4	EMD	CCNL2	
YTHDF1	TAF2	RGS3	PKM	MT1X	HSP90AB1	ELOVL6	CCNL1	
YRDC	TAF15	RFWD2	PKLR	MSN	HSP90AA1	ELOVL4	CCNE1	
YBX3	TADA1	RFPL3	PKIB	MRTO4	HOXA7	ELOVL3	CCNC	
XRCC6	SYNE2	RFC4	PITRM1	MRPS11	HNRNPM	ELAVL1	CCDC9	
XRCC4	SYAP1	REST	PIR	MRPL49	HNRNPA2B1	EIF5A2	CCDC155	
XRCC3	SUPT5H	REM2	PIN1	MPZL1	HNRNPA1	EIF4H	CCDC137	
XPO1	SUMO1	RELA	PIM2	MMS19	HNMT	EIF4A3	CBX5	
XBP1	SULT1C2	REL	PIK3CG	MLH1	HMGN2	EID3	CBX3	
XAGE2	SUDS3	REEP5	PIK3CD	MITF	HMGB1	EGR1	CASK	
WT1	STUB1	RECQL4	PIK3CB	MIS18BP1	HMGA1	EFCAB7	CARM1	
WRNIP1	STRADA	RECQL	PIK3C2A	MED13L	HMBOX1	EEFSEC	CAND1	
WRAP53	STOML2	RBX1	PIGK	MED13	HIST1H4I	EDC3	CAMK2G	

WDR5B	STIP1	RBM8A	PIF1	MED12L	HIST1H3G	E2F4	CAMK1D
WDR5	STAG2	RBM15B	PIAS1	MECOM	HIST1H3F	E2F1	CAMK1
WDR4	SSRP1	RBM14	PHYKPL	MDN1	HIST1H3B	DUSP10	CALML5
VPS18	SSB	RBL2	PHYHD1	MDH1	HIST1H1A	DST	CALD1
VGLL4	SRRM2	RBFA	PHLPP2	MDC1	HIF1A	DPYSL3	CACYBP
VDR	SRP14	RBBP9	PHLPP1	MCRS1	HHAT	DPY30	CABP4
VCP	SRM	RBBP7	PHF1	MCM7	HELZ	DOT1L	C20orf27
VAMP8	SPHK2	RB1CC1	PGS1	MCM5	HDGF	DOLPP1	C1D
VAMP4	SPA17	RAVER1	PGM2	MCM4	HDAC9	DNPH1	C19orf66
VAMP3	SORL1	RARRES2	PGD	MCM3AP	HDAC4	DNMT3B	BZW1
VAMP2	SOAT1	RAD9A	PFKP	MCM3	HDAC3	DNMT1	BRMS1
USP9X	SNRNP70	RAD54L	PEBP1	MCM2	HDAC1	DNAJC8	BRIP1
USP33	SNRNP40	RAD51D	PDS5B	MBD2	HAT1	DNAJC3	BRD2
USP21	SNF8	RAD23B	PDS5A	MAZ	H2AFY2	DNAJC11	BRCA1
UROS	SND1	RAD21	PDK4	MAST1	GTF2A1	DNAJB11	BOD1L1
UPF3A	SMU1	RAD18	PDK3	MAPKAPK5	GSTZ1	DNA2	BMP2K
UPF2	SMG6	RAC1	PDK1	MAPK12	GSS	DMD	BET1L
UPF1	SMG5	RABIF	PDGFRA	MAP7	GSPT1	DIP2C	BDKRB2
UFSP2	SMC6	RABGEF1	PDAP1	MAP4K3	GRWD1	DHX9	BCL11A
UEVLD	SMC5	RAB9A	PCNT	MAP3K4	GRPEL1	DHX40	BAZ2A
UBXN2B	SMC3	RAB6A	PCNP	MAP3K2	GRHL2	DHX38	BARD1
UBTF	SMC1A	RAB5C	PCMT1	MAP2K7	GREM1	DHX37	ATXN2L
UBE2R2	SMARCC2	RAB1B	PCBP1	MALT1	GPS1	DHX34	ATRX
UBE2M	SMARCA2	PUS7	PAXIP1	MADD	GPATCH8	DHX33	ATR
UBE2I	SLX4IP	PURA	PAX5	LYPLA1	GNL3L	DHX16	ATP5F1
UBE2D3	SLTM	PTPN23	PASK	LSM8	GNL3	DHFR	ATP5B
UBE2B	SLC7A9	PTMS	PARP4	LRSAM1	GMIP	DEK	ATP2B1
UBE2A	SLC7A8	PTMA	PARP3	LRRC59	GMDS	DDX50	ATP1A1
UBA1	SLC7A5	PTGES3	PARL	LRRC25	GLI2	DDX24	ATN1
UAP1L1	SLC7A11	PSME3	PAPSS1	LRR1	GIGYF2	DDX1	ATG16L1
UAP1	SLC3A2	PSMD13	PAICS	LPIN2	GIGYF1	DDB1	ATF1
TWF2	SLC25A6	PSMD10	PAFAH1B3	LONP1	GFPT2	DCTN2	ATAD5
TUBE1	SLC25A5	PSMB5	PACSIN3	LIG4	GET4	DCK	ATAD2
TUBB4B	SLC25A36	PSMA2	PACSIN2	LIG3	GDAP1	DBN1	ASF1A
TUBB4A	SKP1	PSKH2	PA2G4	LIG1	GBE1	DBF4B	ARRDC4
TUBB3	SIRT6	PSAT1	OSBPL9	LGMN	GATA6	DAXX	ARRDC3
TUBB2B	SIRT3	PRPSAP2	ORC4	LGALSL	GATA5	DARS	ARPC5
TUBB	SIN3B	PRPS2	OR2H1	LGALS1	GATA4	CTNNA1	ARPC2
TTI1	SIGMAR1	PRPF4B	OGG1	LEMD3	GATA3	CTDP1	ARMC6
TTBK1	SH3BP1	PRPF4	OGFR	LEMD2	GAS2L1	CTBP1	ARL4A
TSPYL5	SFR1	PRPF31	NXNL1	LDHB	GANAB	CTAG2	ARL14
TSPYL4	SFPQ	PRMT5	NVL	LDHA	GAK	CSTF2	ARID4B
TSG101	SF3B4	PRMT2	NUP98	LCP2	FZR1	CSNK2B	ARID4A
TSEN54	SETDB1	PRMT1	NUDT18	LCK	FXN	CSNK2A1	ARID3B
TRPS1	SETD1B	PRKD3	NUDT14	LAGE3	FUS	CSNK1E	ARID3A
TRPC5	SETD1A	PRKD2	NT5DC2	KMT2D	FUBP1	CSE1L	ARID1A
TRIP13	SERPINH1	PRKCSH	NSMCE4A	KMT2C	FRAT2	CRYGS	ARHGDIA
TRIP10	SEPHS2	PRKCQ	NSMCE2	KMT2B	FPGS	CPSF6	ARHGAP42
TRIM28	SENP3	PRKCB	NSFL1C	KMT2A	FOXR1	CPSF4	ARHGAP27
TRIM23	SENP2	PRKAR2A	NRIP1	KLF6	FOXP1	CPNE3	ARF3
TPRKB	SENP1	PRDX3	NR2F2	KLF2	FOXO1	COCH	APPL1
TPR	SEC61A2	PPP6R3	NPM1	KLF17	FOXN2	CNST	APEX1
TP73	SEC61A1	PPP2R1B	NOP9	KLF12	FOXM1	CNPPD1	APEH
TP53RK	SEC24B	PPP2R1A	NOL12	KIF13B	FOXK2	CMTR1	APAF1
TP53BP1	SEC16A	PPP2CA	NOC4L	KIAA1429	FOXJ3	CLPB	AP2A2
TP53	SDHA	PPP1R7	NME4	KIAA0430	FOXF2	CLK3	ANXA4

TOP3B	SCLY	PPP1R1B	NIPSNAP1	KHSRP	FOSL1	CLIC3	ANKLE1
TOP1	SAE1	PPP1R10	NIPBL	KEAP1	FKBP8	CLIC1	ANGPT4
TOMM34	RUVBL2	PPM1G	NFX1	KDM6A	FKBP3	CLASRP	AMPH
TNPO2	RUVBL1	PPM1F	NFE2L3	KDM4B	FHL1	CIRBP	AMPD3
TNKS2	RUNX2	POT1	NFATC2	KDM4A	FES	CHTF18	AMPD2
TNIP2	RTN4	POR	NFATC1	KDM1A	FBXO22	CHN1	ALDH2
TMX1	RTN3	POLR2E	NFAT5	KCTD17	FBP1	CHMP2B	AKT1
TMPRSS13	RTF1	POLR1E	NELFB	KCNS3	FBL	CHEK2	AKR7A3
TMEM109	RTEL1	POLR1C	NEK7	KAT2B	FARSA	CHD8	AKAP8
TLN1	RRAS2	POLR1B	NEK6	KAT2A	FANCM	CGGBP1	AIMP2
TKT	RRAGA	POLR1A	NEK4	JUND	FANCG	CFL1	AIFM1

Supplementary Table 3. Collinearity analysis of seven variables (age, ECOG, LDH level, number of extranodal sites, stage, TRGs risk score and IPI score).

	VIF
Age	1.776
ECOG	1.683
Extranodal_sites	1.316
LDH	2.357
Stage	2.797
RiskScore	1.121
IPI	7.345

Supplementary Table 4. Correlation analysis of seven variables (age, ECOG, LDH level, number of extranodal sites, stage, TRGs risk score and IPI score).

		Age	ECOG	Extranodal sites	LDH	Stage	RiskScore	IPI
Age	Pearson Correlation	1	.155*	−.049	.009	−.030	.164*	.345**
	Sig. (2-tailed)		.026	.480	.902	.668	.019	.000
	N	206	206	206	206	206	206	206
ECOG	Pearson Correlation	.155*	1	.184**	.275**	.266**	.167*	.561**
	Sig. (2-tailed)	.026		.008	.000	.000	.017	.000
	N	206	206	206	206	206	206	206
Extranodal sites	Pearson Correlation	−.049	.184**	1	.103	.374**	−.030	.354**
	Sig. (2-tailed)	.480	.008		.139	.000	.672	.000
	N	206	206	206	206	206	206	206
LDH	Pearson Correlation	.009	.275**	.103	1	.422**	.156*	.658**
	Sig. (2-tailed)	.902	.000	.139		.000	.025	.000
	N	206	206	206	206	206	206	206
Stage	Pearson Correlation	−.030	.266**	.374**	.422**	1	.206**	.703**
	Sig. (2-tailed)	.668	.000	.000	.000		.003	.000
	N	206	206	206	206	206	206	206
RiskScore	Pearson Correlation	.164*	.167*	−.030	.156*	.206**	1	.285**
	Sig. (2-tailed)	.019	.017	.672	.025	.003		.000
	N	206	206	206	206	206	206	206
IPI	Pearson Correlation	.345**	.561**	.354**	.658**	.703**	.285**	1
	Sig. (2-tailed)	.000	.000	.000	.000	.000	.000	
	N	206	206	206	206	206	206	206

*$p < 0.05$; **$p < 0.01$; ***$p < 0.001$.
